# Supplementary material for: Determinants and Sources of Iron Intakes of Australian Toddlers: Findings from the SMILE Cohort Study
Source: Int J Environ Res Public Health. 2019 Jan 9;16(2):181. doi: 10.3390/ijerph16020181 (PMC6352266; doi:10.3390/ijerph16020181)
Supplement: Supplementary file 1 [file ijerph-16-00181-s001.pdf]

Supplementary Table 1. Sensitivity analysis: Factors associated with usual iron intakes (mean values and 95% confidence interval) of toddlers with plausible energy intakes (n=703)

Variables	% below EAR	Unadjusted Mean (mg/d)	95% CI	*p*	Adjusted Mean (mg/d)	95% CI	*p*
Total sample	20.6	6.7	6.5-6.9				
Maternal characteristics							
Maternal age at recruitment (yrs)				0.819			
<25	19.4	6.5	5.7-7.3				
25-34	20.3	6.7	6.5-7.0				
≥35	21.7	6.6	6.2-7.1				
Maternal education - highest level completed				0.196			
High school/vocational	18.3	6.8	6.5-7.2				
Some university and above	22.4	6.6	6.3-6.9				
IRSAD score				0.831			
Deciles 1-2	21.6	6.6	6.0-7.2				
Deciles 3-4	21.2	6.5	6.0-7.0				
Deciles 5-6	20.7	6.7	6.2-7.2				
Deciles 7-8	22.8	6.9	6.4-7.4				
Deciles 9-10	17.2	6.6	6.2-7.1				

Table 3 *Cont.*

Variables	% below EAR	Unadjusted Mean (mg/d)	95% CI	*p*	Adjusted Mean (mg/d)	95% CI	*p*
Maternal country of birth				0.005			0.070
Australia and New Zealand	20.0	6.5	6.3-6.8		6.4	6.2-6.7	
India	23.3	6.5	5.6-7.4		6.6	5.8-7.2	
China	8.6	7.7	6.7-8.7		6.4	5.5-7.2	
UK	20.8	6.2	4.9-7.4		6.1	5.2-7.1	
Asia Other	27.7	8.2	7.4-9.1		7.5	6.8-8.2	
Other	23.7	6.5	5.6-7.5		6.9	6.1-7.7	
Maternal BMI (kg/m^2)				0.935			
<25	21.3	6.6	6.3-6.9				
25-29.99	22.1	6.7	6.2-7.2				
≥30	17.5	6.7	6.1-7.3				
Parity				<0.001			0.009
Primiparous	16.6	7.2	6.9-7.5		6.9	6.5-7.2	
Multiparous	23.8	6.2	5.9-6.6		6.4	6.0-6.8	

Table 3 *Cont.*

Variables	% below EAR	Unadjusted Mean (mg/d)	95% CI	*p*	Adjusted Mean (mg/d)	95% CI	*p*
Child characteristics							
Sex				0.369			
Male	19.8	6.7	6.5-7.1				
Female	21.7	6.6	6.2-6.9				
Primary milk feeding method at 12 months				<0.001			<0.001
Breastmilk	45.6	4.5	4.2-4.8		4.6	4.2-5.0	
Mixed - breastmilk and formula	14.3	7.4	6.7-8.0		7.4	6.7-8.0	
Formula	2.2	8.9	8.6-9.2		9.0	8.6-9.4	
Neither breastmilk nor formula	23.7	5.4	5.0-5.7		5.2	5.2-6.1	

IRSAD, Index of Relative Socio-Economic Advantage and Disadvantage, where decile 1 = most disadvantaged and decile 10 = most advantaged. EAR Estimated average requirement
